# Supplementary material for: The Relationship between Carcass Condemnations and Tail Lesion in Swine Considering Different Production Systems and Tail Lengths
Source: Animals (Basel). 2022 Apr 7;12(8):949. doi: 10.3390/ani12080949 (PMC9030673; doi:10.3390/ani12080949)
Supplement: Supplementary file 1 [file animals-12-00949-s001.zip › animals-1659183-supplementary.pdf]

*animals*

# Supplementary materials

**Table S1.** Requirements descriptions of the three different pig production systems.

| **Requirements** | **Production system** | | |
|---|---|---|---|
| | Conventional[26] | Conventional AB[1] [26]* | Organic [27],[28] |
| Born outdoor/indoor | indoor | | |
| Age at weaning | 21-25 days | | approximately 40 days |
| Tail docking | allowed, but as last resource measure | | prohibited except under specific exceptions** |
| Teeth reduction | grinding or partially cut only if necessary, within the first 3 days | | |
| Resting area with bedding | required | | |
| Floors | slat/solid floor | | slat/solid floor - at least 50% of the required minimum area should have solid floor |
| Access to an outdoor facility | optional | | required |
| Batch mixing | allowed | not allowed | |
| Area required per pig | 0.65 $m^2$/head | | indoor area - 1,1$m^2$/head; outdoor area – 1$m^2$/head |
| Access to roughage | optional | | required |
| Treatment with antibiotics | allowed | prohibited from 65 days of life | prohibited*** |
| Withdrawal time related to veterinary administrations | if the medication does not indicate the withdrawal time, it has got be at least 28 days | twice as long as the legislation indicates (if the time indicated by the medication is too short - at least 48h) | |

[1] Antibiotic-free
* Conventional antibiotic-free is a commercial category established by the producing company
**Although most of the organically raised animals in this study presented docked tails, this is stated in the European regulation.
***Declassified as organic pig if: 3 or more treatments with antibiotics, within 12 months OR 1 or more treatment if its productive life cycle is less than one year.


[26] Ministerio de Agricultura Pesca y Alimentación. Royal Decree 1135/2002 of October 31, Relative to the Minimum Standards for the Protection of Pigs. *Boletin Oficial Del Estado* **2002**, 1–8.

[27] The Council of the European Union. Council Regulation (EC) No 834/2007 of 28 June 2007 on Organic Production and Labelling of Organic Products and Repealing Regulation (EEC) No 2092/91. *Official Journal of the European Union* **2007**, *L 189*, 1–23.

[28] The Commission of the European Communities. Commission Regulation (EC) No 889/2008 of 5 September 2008 Laying down Detailed Rules for the Implementation of Council Regulation (EC) No 834/2007 on Organic Production and Labelling of Organic Products with Regard to Organic Production, Labelling and Control. *Official Journal of the European Union* **2008**, *L 250*, 1–84.

**Table S2.** Values (mean ± Standard Error and range) of batch-level (*n* = 73) tail lesions and scarring scores by production system and tail-docking practices in pigs' batches examined at the slaughterhouse.

| | | Batch tail lesion score | | Batch tail scarring score | |
|---|---|---|---|---|---|
| | N | Mean ± SE | Range (min – max) | Mean ± SE | Range (min – max) |
| All batches | 73 | 0.85 ± 0.03 | 0.26 – 1.74 | 0.17 ± 0.02 | 0 – 0.66 |
| **Production System** | | | | | |
| Conventional | 51 | 0.87 ± 0.04 | 0.37 – 1.74 | 0.18 ± 0.02 | 0 – 0.66 |
| Conventional AB[1] | 12 | 0.82 ± 0.06 | 0.55 – 1.22 | 0.14 ± 0.03 | 0 – 0.34 |
| Organic | 10 | 0.75 ± 0.10 | 0.26 – 1.12 | 0.13 ± 0.03 | 0 – 0.31 |
| **Tail-docking** | | | | | |
| Fully docked | 57 | 0.80 ± 0.03 | 0.26 – 1.39 | 0.16 ± 0.02 | 0 – 0.66 |
| Docked at mid-length | 7 | 0.88 ± 0.07 | 0.64 – 1.12 | 0.16 ± 0.03 | 0.06 – 0.31 |
| Undocked | 9 | 1.10 ± 0.10 | 0.76 – 1.74 | 0.19 ± 0.04 | 0.04 – 0.42 |

[1] Antibiotic-free

**Table S3.** Logistic regression model exploring batch-level variation in the occurrence of total condemnations due to pyaemia in pigs' batches (*n* = 73) at the slaughterhouse. For significant variables, odds ratio estimates (OR) and their 95% confidence interval (CI) are presented, with estimates for continuous scores calculated for a 0.5 unit increase. Significant p-values and ORs are highlighted in bold.

| Response variable | Explanatory variable | Statistic | *p*-Value | Odds Ratio | |
|---|---|---|---|---|---|
| | | | | Estimate | 95%CI |
| TC[1] due to pyaemia | Batch tail lesion score | $\chi^2_1$=6.22 | **0.0126** | **2.06** | **1.16 – 3.63** |
| | Batch scarring score | $\chi^2_1$=13.79 | **0.0002** | **3.86** | **1.89 – 7.88** |
| | Production system | $\chi^2_2$=2.30 | 0.32 | | |
| | Tail length | $\chi^2_2$=0.45 | 0.80 | | |

[1] Total condemnations

**Table S4.** Logistic regression model exploring batch-level variation in local condemnation due to abscesses probability within pigs' batches (*n* = 73) at the slaughterhouse. For significant variables, odds ratio estimates (OR) and their 95% confidence interval (CI) are presented, with estimates for continuous scores calculated for a 0.5 unit increase. Significant p-values and ORs are highlighted in bold.

| Response variable | Explanatory variable | Statistic | *p*-Value | | Odds Ratio Estimate | Odds Ratio 95%CI |
|---|---|---|---|---|---|---|
| LC[1] due to abscess | Batch tail lesion score | $\chi^2_1$=0.50 | 0.48 | | | |
| | Batch scarring score | $\chi^2_1$=44.69 | **<0.0001** | | **3.65** | **2.50 – 5.34** |
| | Production system | $\chi^2_2$=2.01 | 0.37 | | | |
| | Tail length | $\chi^2_2$=17.24 | **0.0002** | Undocked vs Fully docked | **1.70** | **1.13 – 2.57** |
| | | | | Undocked vs Docked at mid-length | 0.81 | 0.49 – 1.33 |
| | | | | Docked at mid-length vs Fully docked | **2.10** | **1.43 – 3.10** |

[1] Local condemnations
